# Plasma endothelin-1 levels are increased in atrial fibrillation patients with hyperthyroidism

**DOI:** 10.1371/journal.pone.0208206

**Published:** 2018-12-04

**Authors:** Fadia Mayyas, Nesreen Saadeh, Kusai Al-Muqbel, David R. Van Wagoner

**Affiliations:** 1 Department of Clinical Pharmacy, Faculty of Pharmacy, Jordan University of Science and Technology, Irbid, Jordan; 2 Department of Internal Medicine, Faculty of Medicine, Jordan University of Science and Technology, Irbid, Jordan; 3 Department of Diagnostic Radiology and Nuclear Medicine, Faculty of Medicine, Jordan University of Science and Technology, Irbid, Jordan; 4 Department of Molecular Cardiology, Cleveland Clinic, Ohio, United States of America; Rush University Medical Center, UNITED STATES

## Abstract

**Background:**

Endothelin-1 (ET-1) is a potent vasoconstrictor, mitogen and inflammatory factor that may contribute to development of atrial fibrillation (AF). Plasma ET-1 levels are increased in hyperthyroid patients, but studies evaluating its relation to AF development in hyperthyroid patients are lacking.

**Objective:**

The present study seeks to evaluate the relation of plasma ET-1 to AF development as a function of thyroid status.

**Methods:**

Blood samples from euthyroid patients (n = 41), hypothyroid (n = 61), hyperthyroid (n = 41), AF with hyperthyroidism (n = 9), and euthyroid AF (n = 10) patients were collected. Plasma ET-1, CRP, and thyroid hormone levels were measured and compared between groups.

**Results:**

Plasma ET-1 levels were higher in hyperthyroid and euthyroid AF patients> hyperthyroid-non-AF > hypo and euthyroid non-AF patients. Plasma ET-1 levels positively correlated with free T3 and T4 levels, and negatively with TSH levels. By multivariate analysis, plasma ET-1 was positively associated with AF, hyperthyroidism, and age. Plasma CRP did not vary by study group in either univariate or multivariate analyses.

**Conclusion:**

Plasma ET-1 is associated with AF, elevated in hyperthyroid patients and positively correlated with thyroid hormone levels, suggesting that hyperthyroidism may increase ET-1 expression and release. This study may guide development of novel predictors of AF associated with hyperthyroidism, and may help to personalize therapy in hyperthyroid patients.

## Introduction

Hyperthyroidism is common, affecting older people more than younger, and women more than men [1]. Graves’ disease is the most common cause of hyperthyroidism. Other significant causes include toxic thyroid nodules and early phase subacute thyroiditis [1]. Hyperthyroidism is associated with long-term cardiovascular sequelae that include increased risk of atrial fibrillation (AF) [2], the most common form of cardiac arrhythmia. AF is associated with major complications including thrombosis, stroke, heart failure (HF) and death [2]. Interestingly, patients with undetectable thyroid stimulating hormone (TSH) levels have a 3-fold higher risk of AF versus those with normal TSH levels [3]. The mechanisms linking hyperthyroidism to AF are not yet fully understood.

Endothelin-1 (ET-1) is a potent vasoconstrictor and mitogen that promotes inflammation, oxidative stress, cardiac myocyte hypertrophy and interstitial fibrosis. These pathways can create a substrate that promotes AF development and progression [4, 5]. We and others have shown that the abundance of ET-1 in the atria and in plasma is increased in AF patients [4, 6–8] and in experimental models of AF [9]. Interestingly, plasma ET-1 is also increased in patients and animal models of hyperthyroidism [10–14]. Thyroid hormone T3 is reported to induce upregulation of ET-1 mRNA and protein via a PKC-α dependent pathway [15]. Increased plasma ET-1 might also reflect endothelial dysfunction caused by high thyroid hormone levels [10].

In a preclinical study in hyperthyroid rats, use of an endothelin antagonist reduced ET-1 expression [13]. Use of methimazole, an antihyperthyroid drug, decreased plasma levels of ET-1 and thyroid hormones in hyperthyroid patients [10], suggesting that ET-1 expression is modulated by thyroid function. However, the relation of elevated ET-1 to risk for AF in hyperthyroid patients is unclear. To better understand this relationship, our study evaluated plasma levels of ET-1 in euthyroid, hyperthyroid, and hypothyroid patients with and without a history of AF.

## Methods

All studies were performed with written informed consent and approval from the Institutional Review Board of King Abdullah University Hospital and Jordan University of Science and Technology.

Adult patients (18 years or older) who presented to the endocrinology clinic of King Abdullah University Hospital (KAUH) with hyper-, hypo- or euthyroid status were included. Patients with either clinical or subclinical hyperthyroidism have an increased risk of AF [3] and both groups were included in this study. AF patients were referred from the cardiology department to endocrinology outpatient clinic when hyperthyroidism was suggested as the underlying cause of AF. Similarly, patients who presented to the endocrinology clinic with hyperthyroidism and hyperthyroidism with AF were referred to the cardiology clinic for confirmation. AF was documented based on electrocardiographic findings recorded by surface ECG. Euthyroid, hypothyroid and hyperthyroid patients with previous history of AF were excluded in order to guarantee selection of AF that is likely secondary to hyperthyroidism. Patient samples were categorized into four groups based on thyroid status and arrhythmia history.

Patient demographic information, clinical characteristics, medication use and biochemical data (eg., free total triiodothyronine, T3; thyroxine, T4; and thyroid stimulating hormone, TSH) and use of medications were obtained by query of the patients’ electronic medical record (chart review) at the outpatient clinics KAUH.

### Blood collection

Blood samples were collected at KAUH clinical labs for measurement of thyroid hormones and plasma biomarkers at time of presentation. Freshly drawn blood samples were transferred on ice and centrifuged at 700 x *g* for 15 minutes to separate cells from plasma. EDTA-plasma aliquots were stored at -80°C until analysis.

### Plasma ET-1 measurements

Plasma ET-1 levels were measured using a colorimetric enzyme-linked immunosorbent assay (Endothelin-1 Quantikine ELISA Kit, R&D Systems, USA & Canada). Briefly, 75 μL of standard or sample was added to each microplate well coated with ET-1 primary antibody and incubated for 1 hour on a shaker at room temperature. Following washing, 200 μL of ET-1 conjugate was added to each well and incubated for 3 hours at room temperature on the shaker. After washing, wells were incubated with 200 μL of substrate solution followed by addition of 50 μL of stop solution. Optical density of each well was determined at 450 nm using an Epoch Biotek microplate reader (BioTek, Winooski, VT, USA).

### Plasma C-reactive protein (CRP) measurements

Plasma CRP levels were measured using the Human C-reactive protein/CRP Quantikine ELISA kit (R&D Systems, USA & Canada) per manufacturer’s directions. Wells were coated with CRP capture antibody and incubated overnight at room temperature.

Following washing and blocking, a 100 μL of sample or standard was added and incubated for 2 hours at room temperature. Next, 100 μL of the detection antibody was added. After washing, a 100 μL of streptavidin-horseradish peroxidase (HRP) solution was added to each well and incubated for 20 minutes at room temperature. After washing, wells were incubated with 100 μL of substrate solution followed by addition of 50 μL of stop solution. The optical density of each well was determined at 450 nm using an Epoch Biotek microplate reader (BioTek, Winooski, VT, USA)

### Statistical analysis

Data are expressed as mean ± standard error, unless otherwise specified. Univariate analyses were performed first to evaluate the association of clinical and demographic variables with plasma ET-1 levels. Data were analyzed using Analysis of variance (ANOVA) for normally distributed variables and Kruskal Wallis test for non-normally distributed variables. Chi-square tests were used to compare frequencies across study groups. Pearson correlation was used to evaluate correlations between variables. Univariate analyses and figures were performed using Graph Pad Prism 6. Multivariate analysis adjusting for possible confounders (including patient characteristics and medication use) was performed to evaluate independent predictors of plasma ET-1 and CRP levels in a stepwise manner using JMP 11 software (SAS, USA). Only variables with P<0.3 were kept in the final model. Shapiro-Wilk normality test was used to test data distribution. Because plasma levels of ET-1, CRP and thyroid hormones were not normally distributed, a log transformation was performed. The log transformation effectively normalized the data. Values of p<0.05 were considered statistically significant.

## Results

### Patient characteristics

Plasma samples from 162 patients were selected for analysis of plasma ET-1, CRP and other lab measurements (thyroid hormones, etc.). Table 1 shows the study groups stratified based on thyroid and AF status. Study groups included: 1) control euthyroid patients with sinus rhythm (control, N = 41); 2) hypothyroid patients with sinus rhythm (Hypo, N = 61); 3) Hyperthyroid patients with sinus rhythm (Hyper, N = 41); 4) AF with hyperthyroidism (Hyper+AF, N = 9); and euthyroid patients with AF (AF, N = 10). None of the hyperthyroid patients had a history of AF, and hyperthyroidism was documented as the likely cause of AF in 9 patients who presented in AF. Ten euthyroid patients who presented in AF were included for comparison. Most study patients were female (74.1%) with an average BMI of 28.9±0.50 kg/m^2^. Mean age±SEM of patients was 43.3±1.2 years and the euthyroid AF group was significantly older than other groups (Table 1). Diabetes mellitus (DM) and hypertension (HT) were present in 21.8% and 30.9% of patients. 27 patients (16.7%) were smokers and 14 had coronary artery disease (CAD, 8.6%). 4 and 16 patients had subclinical hyper- and hypothyroidism; respectively. Among the patients with clinical hyperthyroidism, 20 had Graves’ disease, 2 had toxic adenoma, 5 toxic multi-nodular goiters, 6 early phase subacute thyroiditis, 1 operated papillary thyroid cancer, 3 excess levothyroxine hormone replacement for chronic thyroiditis intake and in 9 patients the cause was undocumented. About 18% of hypothyroid patients were on levothyroxine and 8% of all hyperthyroid patients were on carbimazole.

**Table 1:** Baseline patient characteristics.

	Euthyroid N = 41	Hypo N = 61	Hyper N = 41	Hyper+AF N = 9	AF N = 10	P value
Age	42.46±2.11	43.09±1.84	39.60±2.23	46.44±6.76 444±6.76*	60.60±5.15*	<0.0185
Male gender	9 (21.9)	9 (14.7)	13 (31.7)	5 (55.6)	6 (60.0)	0.0044
BMI	29.57±0.76	31.25±0.78	25.53±1.01*	25.27±2.85*	28.98±1.65	<0.0001
HT	12 (30.0)	17(27.8)	7 (17.1)	4 (44.4)	9 (90.0)	0.0003
DM	15 (36.6)	11 (18.0)	5 (12.2)	1 (11.1)	3 (30.0)	0.0602
Smoking	8 (19.5)	4 (6.5)	10 (24.4)	3 (33.3)	2 (20.0)	0.0790
CAD ≥50% stenosis,	3 (7.3)	2 (3.2)	1 (2.4)	2 (22.2)	6 (60.0)	<0.0001
Free T3	4.50±0.17	3.83±0.14	13.13±1.94*	9.07±1.99	4.88±0.18	<0.0001
Free T4	15.02±0.43	10.44±0.60	36.86±4.32*	27.86±5.16	15.54±0.75	<0.0001
TSH	2.48±0.22	17.52±3.31	0.077±0.04*	0.017±0.006	3.26±0.45	<0.0001
Carbimazole	0 (0.0)	0 (0.0)	3 (7.3)	1(11.1)	0 (0.0)	0.0472
Levothyroxine	0 (0.0)	11 (18.0)	0 (0.0)	0 (0.0)	0 (0.0)	0.0006
Beta blocker	12 (29.3)	8 (13.1)	14 (34.1)	5 (55.6)	4 (40.0)	0.0187
ACEi/ARB	9 (69.2)	6 (9.8)	3 (7.3)	2 (22.2)	2 (20.0)	0.2423
Aspirin	5 (12.2)	8 (13.1)	3 (7.3)	3 (33.3)	7 (70.0)	<0.0001
Statins	9 (21.9)	5 (8.2)	4 (9.7)	2(22.2)	4 (40.0)	0.0401

Data are presented as mean±SEM for continuous variables and % for categorical variables. BMI: body mass index (kg/m^2^); HT: hypertension; DM: diabetes mellitus; CAD: coronary artery disease; ACEi: angiotensin converting enzyme inhibitor; ARB: angiotensin receptor blocker. Unit for serum T3, T4 and TSH is pmol/L.

*indicates presence of significant differences relative to control (p<0.05). P-values represent ANOVA, Kruskal, or Chi square tests’ probability of difference between study groups

### Univariate predictors of plasma ET-1 and CRP

Table 2 documents predictors of plasma ET-1 and CRP.

**Fig 1:**
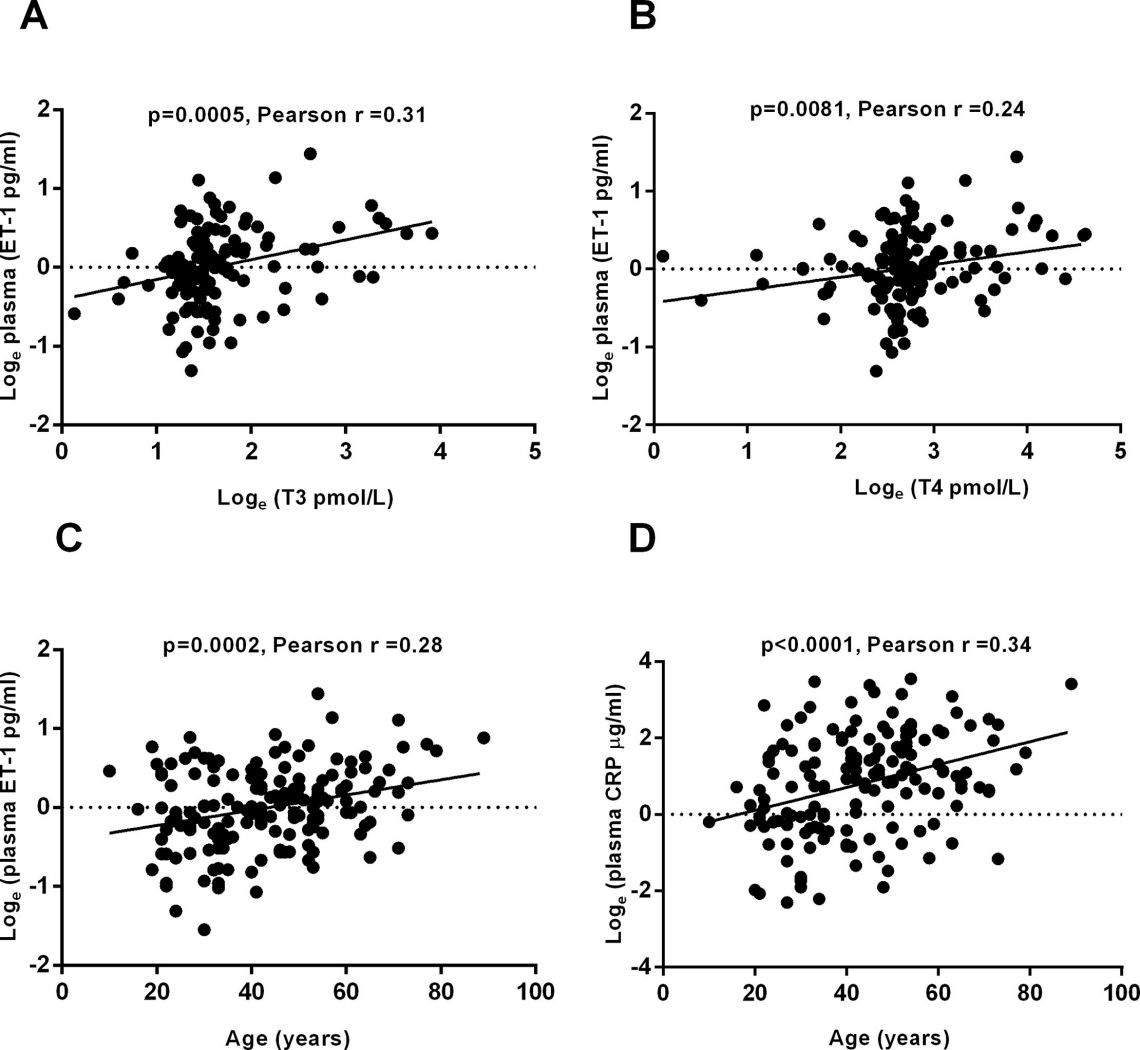
Correlation of plasma ET-1 and CRP with thyroid hormones and age. Fig 1 shows the relationship of log plasma ET-1 with age (A), log T3 (B), and log T4 (C). Fig 1D shows the relationship of the log plasma CRP with age.

Plasma ET-1 levels were positively associated with age (Fig 1A), male gender, smoking history, serum T3 and T4 (Fig 1B–1C), hyperthyroidism, euthyroid AF, AF associated with hyperthyroidism, HT, CAD, and use of aspirin, beta blockers, and carbimazole. Hypothyroidism and serum TSH were negatively correlated with plasma ET-1 (Table 2). Plasma CRP was positively correlated with age (Fig 1D), BMI, HT, systolic blood pressure and DM (Table 2).

**Table 2:** Univariate predictors of plasma ET-1 and CRP.

Response = log plasma ET-1 or CRP				
N = 162 Plasma ET-1			Plasma CRP	
	P value	Beta	P value	Beta
Age, yrs.	0.0002*	3.75	<0.0001*	4.56
Gender, Male	0.0002*	3.80	0.6952	0.39
BMI	0.0595	-1.9	0.0002*	3.80
Smoking	0.0154*	2.45	0.2336	-1.19
Hypertension	0.0134*	2.50	0.0025*	3.07
Systolic blood pressure, mmHg	0.1960	1.3	0.0127*	2.53
Diastolic blood pressure, mmHg	0.2125	1.25	0.0894	1.71
CAD ≥50%	0.0042*	2.91	0.1519	1.44
Diabetes	0.7569	-0.31	0.0094*	2.63
Hypothyroidism	0.0015*	-3.22	0.9175	-0.10
Hyperthyrodism	0.0111*	2.57	0.3053	-1.02
AF+Hyperthyroidism	0.0003*	3.70	0.1869	-1.30
AF+Euthyroidism	<0.0001*	4.36	0.4137	0.82
Log T3	0.0045*	2.90	0.8795	0.15
Log T4	0.0081*	2.69	0.4523	-0.75
Log TSH	0.0004*	-3.6	0.7691	0.29
Carbimazole	0.0266*	2.24	0.5328	-0.62
Levothyroxine	0.3115	-1.02	0.7934	0.26
Beta Blocker	0.0184*	2.38	0.7021	-0.38
ACEi/ARB	0.3002	-1.04	0.0524	1.95
Aspirin	0.0001*	3.35	0.2564	1.13
Statin	0.1367	1.49	0.3096	1.02

BMI: body mass index Kg/m^2^; CAD: coronary artery disease; AF: atrial fibrillation; T3: free total triiodothyronine; T4: thyroxine; TSH: thyroid stimulating hormone; ACEi/ARB: angiotensin converting enzyme inhibitor or angiotensin receptor blocker. Beta is the standardized coefficient (slope/std error).

*indicates presence of significant differences (p<0.05).

**Fig 2:**
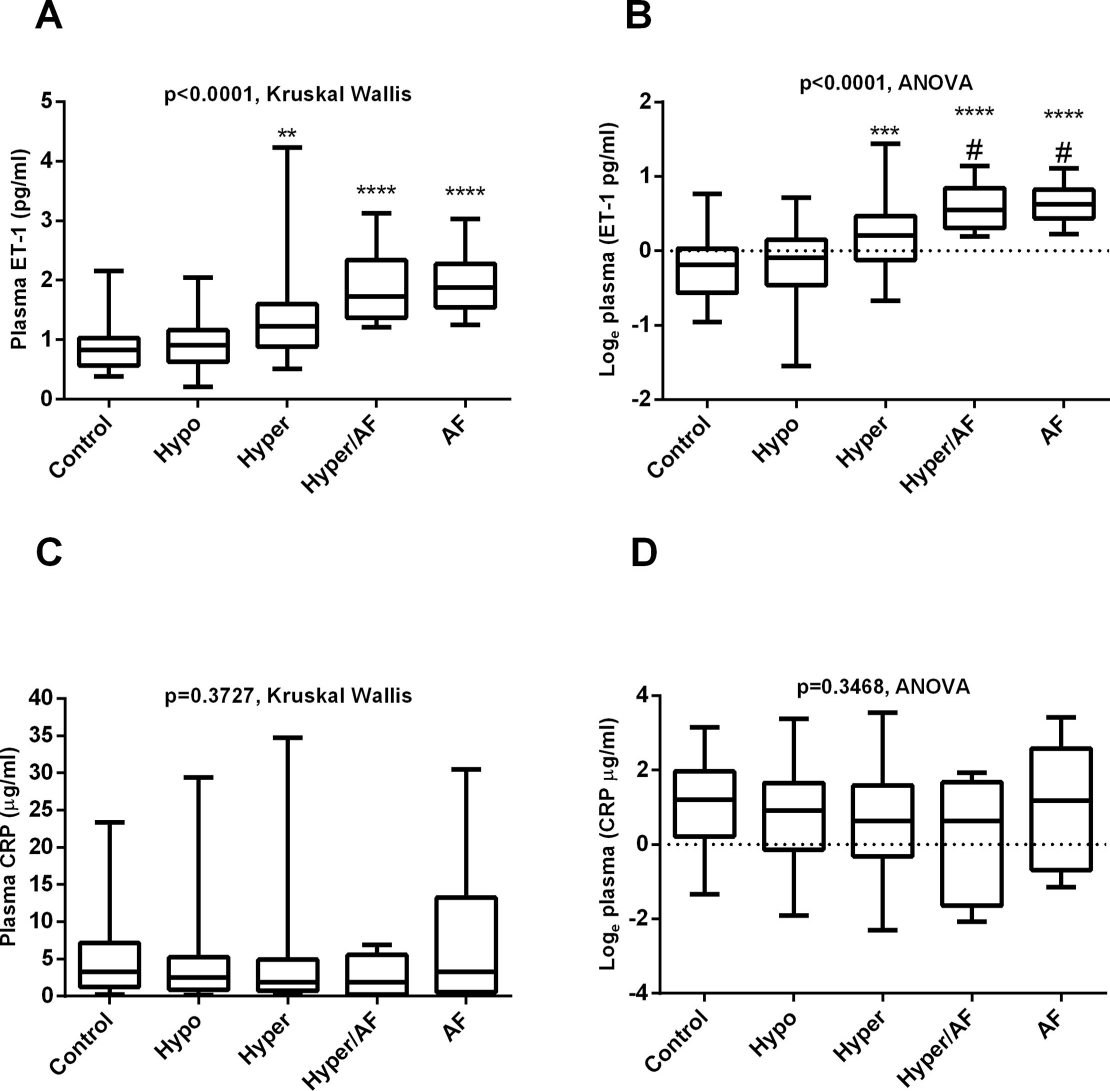
Relation of plasma ET-1 and CRP to thyroid status. Fig 2 represents plasma levels of ET-1 (A) and log transformation (B) among control, hypothyroid, hyperthyroid with sinus rhythm or atrial fibrillation (AF) associated with hyperthyroidism, and in euthyroid AF patients. Fig 2C–2D represent changes in plasma CRP before and after log transformation in the same groups.

Fig 2 shows the plasma levels of ET-1 before (A) and after log transformation (B). Plasma ET-1 levels were significantly increased in both AF and hyperthyroid patients (mean±SEM: 1.96±0.164, 1.89±0.207, 1.31±0.101, 0.949±0.055, and 0.871±0.060 pg/ml for AF, Hyper+AF, Hyper, Hypo, and control; respectively, p<0.0001, Fig 2A). Plasma ET-1 levels tended to be higher among those with hyper- and euthyroid AF patients than hyperthyroid patients with sinus rhythm, (Fig 2B). No differences in plasma CRP were detected between study groups as a function of thyroid status (Fig 2C–2D).

### Multivariate predictors of plasma ET-1

By stepwise analysis, gender, BMI, HT, DM, CAD and use of beta blockers or carbimazole were not significantly associated with the log plasma ET-1 and were excluded from the model. By step wise analysis, T3, T4 and TSH were significantly and independently associated with plasma ET-1, however, they were excluded from the model to avoid collinearity with hyperthyroidism status. After adjusting for other variables, log plasma ET-1 was significantly and independently associated with age, hyperthyroidism, AF associated with hyperthyroidism, and euthyroid AF (all p<0.05, Table 3).

**Table 3:** Multivariate independent predictors of plasma ET-1.

Response = log of plasma ET-1 N = 162				
Patients characteristics	Estimate (slope, B)	Standard error	P value	Beta
Age, yrs.	0.0062	0.0026	0.01971*	2.41
Smoking	0.0827	0.04676	0.0788	1.77
Hypothyroidism	0.0338	0.04309	0.4345	0.78
Hyperthyrodism	0.3014	0.0473	<0.0001	4.26
AF+Hyperthyroidism	0.3665	0.0791	<0.001*	4.63
AF+Euthyroidism	0.3583	0.0831	<0.0001*	4.31
Aspirin	0.0618	0.0546	0.2588	1.13

AF: atrial fibrillation. Beta is the standardized coefficient (slope/standard error).

*indicates presence of significant differences (p<0.05).

We also performed a logistic regression analysis to study the predictors of AF in patients, and found that hyperthyroidism, HT, CAD and male gender are significantly and independently associated with AF risk independent of age (Table 4, model A). Because plasma ET-1 was collinear with hyperthyroidism, it was not included in this model. However, in another model excluding hyperthyroidism, plasma ET-1 was also significantly associated with AF independent of age or CAD (Table 4, model B).

**Table 4:** Predictors of atrial fibrillation.

Response = atrial fibrillation, N = 162				
	Model A		Model B	
Patients characteristics	P value	Odd ratio	P value	Odd ratio
Age, yrs.	0.8405	0.96	0.3729	0.96
Male Gender	0.0123*	4.59	0.1733	2.59
Hypertension	0.0326*	6.22	0.1014	4.63
CAD	0.0018*	10.99	0.0062*	12.34
Hyperthyroidism	0.0235*	4.49	-	-
Log ET-1	-	-	0.0003*	69.24

CAD: coronary artery disease, ET-1: endothelin 1.

*indicates presence of significant differences relative (p<0.05).

### Multivariate predictors of CRP

By stepwise analysis, BMI, HT, serum TSH, T3 and T4 were not correlated with log plasma CRP and were excluded from the model. By multivariate analysis, plasma CRP levels were significantly and independently associated only with age and smoking (p<0.05). Adjusting for potential confounders, plasma CRP was not associated with thyroid status (Table 4). Statin use was marginally associated with the log plasma CRP (Table 5).

**Table 5:** Independent predictors of plasma CRP.

Response = log plasma CRP				
Patients characteristics	Estimate (slope, B)	Standard error	P value	Beta
Age, yrs.	0.0347	0.6603	<0.0001*	4.43
Gender, Male	0.1762	0.1425	0.2184	1.24
Smoking	0.3633	0.1668	0.0310*	2.18
Hypothyroidism	-0.1887	0.1312	0.1522	1.44
Hyperthyrodism	-0.1588	0.1419	0.2646	1.12
Hyperthyroidism+AF	-0.4323	0.2472	0.0824	1.75
AF	-0.3466	0.2621	0.1881	1.32
Diabetes	0.1563	0.1351	0.2491	1.16
Use of Statin	-0.2900	0.1631	0.0775	-1.78

AF: atrial fibrillation. Beta is the standardized coefficient (slope/standard error).

*indicates presence of significant differences (p<0.05).

## Discussion

Hyperthyroidism is associated with profound cardiovascular complications such as myocardial infarction and AF in patients [2, 3, 8, 16] and in animal models [17]. The most common cardiac arrhythmias encountered in hyperthyroid patients are sinus tachycardia and AF. About 10–15% of hyperthyroid patients develop AF [18]. AF is associated with increased risk of thrombosis, stroke, and mortality [4].

The mechanism(s) whereby thyroid disorders promote AF are still not fully understood. In a rat thyroidectomy model, both hypo- and hyperthyroid status promoted AF, via distinct mechanisms that include changes in ion channel expression (prominent in hyperthyroidism), and structural remodeling that includes changes in cardiac function, myocyte size and interstitial fibrosis (prominent in hypothyroidism) [19].

Previous studies documented the role of ET-1 on the substrates of AF in patients with underlying cardiac disease [4, 20, 21]. AF may develop secondary to hyperthyroidism, however, the mechanisms by which hyperthyroidism promotes AF development are not clear. This study links for the first time the significance of elevated ET-1 in hyperthyroid patients with risk of AF that is likely due to hyperthyroidism.

Plasma ET-1 is increased in hyperthyroid patients [10, 11] and increased expression of ET-1 within the atria promotes atrial remodeling, inflammation and AF [4–6, 17]. Increased activity of the stress response c-jun N-terminal kinase 2 (JNK2) in the aged atria may promote arrhythmic remodeling by increasing expression and activity of Ca^2+^/calmodulin-dependent kinase II delta and Ca^2+^ release from the sarcoplasmic reticulum [22, 23]. Similar to our previous studies [4, 24], plasma ET-1 levels were also elevated with increasing age and smoking. ET-1 is a G protein coupled receptor that promote cardiac remodeling via activation of Erk1/2 of the MAPK pathway [25]. Our current and previous findings [4, 24] document increased ET-1 in older patients, suggesting that elevated ET-1 in aged patients may increase JNK2 crosstalk with CaMKII and promote atrial remodeling and arrhythmogenesis. Interestingly, use of ethanol causes a reduction in coronary artery smooth muscle cell potassium currents and coronary contractility via the MAPK pathway, and use of ET-1 amplified the contractile responses to ethanol [26]. Proteomic analysis identified ERK1/2, MAPKs, and Akt as ET-1 targets and key mediators of cardiac myocyte proliferation and terminal differentiation [27]. Together, these finding suggest that elevated ET-1 that is associated with aging may activate key components of the MAPK pathway and promote atrial remodeling that underlies atrial rhythmogenesis.

In our study, euthyroid AF patients were significantly older than patients in other groups raising a possible confounding effect of age. AF is commonly associated with aging and with presence of underlying cardiac disease. Most of our euthyroid AF patients had CAD which might contribute to AF development. By multivariate analysis, both AF and hyperthyroidism were associated with increase plasma ET-1, independent of age. In further analysis, we confirmed that both increased plasma ET-1 and hyperthyroidism were associated with AF risk, independent of age or CAD.

Here, we confirmed that plasma ET-1 levels are elevated in hyperthyroid patients and demonstrated that they are independently associated with increased risk of AF. Interestingly, ET-1 inhibits voltage gated K^+^ currents (via Kv1.5 encoded pore subunits) in pulmonary artery myocytes [28] that are also abundantly expressed in human atrial myocytes, and which are present but downregulated in the atria of patients with AF [29]. ET-1 can directly and indirectly contribute to alterations in calcium cycling [29] that may contribute to increased AF risk.

Plasma abundance of ET-1 was higher in euthyroid AF patients and in AF associated with hyperthyroidism than in hyperthyroid patients without AF. This suggests an additive or synergistic impact of hyperthyroid status and AF on plasma ET-1 abundance, and that the marked increase of ET-1 may contribute to AF development or persistence in hyperthyroid patients. That our AF patients presented in AF rhythm may explain their slightly higher ET-1 levels, as elevated wall stress promotes ET-1 expression and release [4]. This observation is likely to be clinically relevant, as biomarker assessment of ET-1 may improve AF risk prediction and open new avenues for personalized targeted therapies in hyperthyroid patients.

Plasma ET-1 levels were positively correlated with free T3 and T4 levels, suggesting that, as in mice [17], thyroid hormones may stimulate ET-1 production in hyperthyroid patients. Hyperthyroidism is a potent stimulus for myocardial hypertrophy, as a result of its effects on contractility, heart rate and metabolism, and by the direct actions of T3 on cardiomyocytes [30]. Interestingly, mice with specific disruption of the cardiac ET-1 gene are resistant to thyroid hormone-induced myocyte hypertrophy, suggesting that ET-1 may act as a mediator of cardiovascular hypertrophy in hyperthyroid rats [17]. Atrial remodeling and hypertrophy are important substrates for development of atrial fibrillation. We have previously found that atrial ET-1 expression is closely correlated with atrial hypertrophy, dilation and fibrosis [4].

Further evidence that AF and other tachyarrhythmias may promote ET-1 production is provided by the observation that plasma ET-1 levels drop quickly after catheter ablation [7]. Thyroid hormones have been suggested to promote paroxysmal AF via increased triggered activity in the pulmonary veins [31]. In AF patients, treatment of hyperthyroidism facilitates conversion to sinus rhythm in two-thirds of patients [18]. Use of methimazole, an anti-hyperthyroid drug, reduced plasma levels of ET-1 and thyroid hormones [10]. Moreover, use of endothelin antagonist reduced the increase in ET-1 and ET-1 converting enzymes in hyperthyroid rats [13]. This observation may lead to development of novel and safer therapeutic approaches that selectively target the ET-1 system to alter the progression of AF in hyperthyroid patients.

Previous studies found a close correlation of plasma ET-1 with the increase in thyroid metabolic activity independent of the cause, suggesting that plasma ET-1 may serve as a useful functional index of thyroid activity (10–11). We also found that plasma ET-1 was independently and positively correlated with free plasma T4 and T3 levels, suggesting that metabolic changes in hyperthyroidism may promote ET-1 expression or release.

CRP is a sensitive but non-specific marker of systemic inflammation that is associated with atherosclerotic cardiovascular disease risk [32]. Plasma CRP has been reported to increase as function of AF persistence [33] and to predict future development of AF [34]. Here, we measured plasma CRP as a control biomarker of inflammation to evaluate whether changes in plasma ET-1 are specific for AF secondary to hyperthyroidism. In our study patients, plasma CRP levels were not associated with thyroid status [35]. However, plasma CRP was associated with increased age and smoking.

### Strengths and limitations of the study

To the best of our knowledge, this is the first study to evaluate the relationship of plasma ET-1 levels with AF status in hyperthyroid patients. Due to the low prevalence of AF secondary to hyperthyroidism, relatively few patients were available, limiting the power of this analysis. AF patients with sinus rhythm were older than other groups and most of them had CAD which might contribute to AF development. Future studies should include age-match patients without CAD to avoid potential confounding.

### Future recommendations

This study is an association study that does not prove a causal relationship. However, it provides new insights regarding the important role of ET-1 in the etiology of AF, particularly in those with hyperthyroidism. Future follow-up studies are recommended in order to assess the utility of high baseline plasma ET-1 to predict AF development or persistence in patients with different underlying pathologies. It would be of interest also to test the relation of follow-up ET-1 levels to AF persistence among hyperthyroid AF patients after conversion to euthyroidism.

## Conclusion

Plasma ET-1 levels are associated with AF development in hyperthyroidism and are positively correlated with thyroid hormones suggesting that metabolic changes in hyperthyroidism may modulate ET-1 expression. This study may facilitate development of novel predictors of AF secondary to hyperthyroidism. Further research is recommended to investigate the potential utility of novel therapeutic agents that decrease plasma ET-1 to reduce the burden of AF in patients.

## Supporting information

S1 Dataset S1 dataset represents an excel file that includes data used to generate figures and tables of the study. (XLSX)
